# Blood Compatibility of Hydrophilic Polyphosphoesters

**DOI:** 10.1021/acsabm.1c01210

**Published:** 2022-02-24

**Authors:** Chiara Pelosi, Iren Constantinescu, Helena H. Son, Maria Rosaria Tinè, Jayachandran N. Kizhakkedathu, Frederik R. Wurm

**Affiliations:** † Dipartimento di Chimica e Chimica Industriale, Università di Pisa, Via Moruzzi 13, 56120 Pisa, Italy; ‡ Center for Blood Research, Life Sciences Centre, Department of Pathology and Laboratory Medicine, University of British Columbia, 2350 Health Sciences Mall, Vancouver, British Columbia V6T 1Z3, Canada; § School of Biomedical Engineering, University of British Columbia, 2350 Health Sciences Mall, Vancouver, British Columbia V6T 1Z3, Canada; ∥ Sustainable Polymer Chemistry (SPC), Department of Molecules and Materials, MESA+ Institute for Nanotechnology, Faculty of Science and Technology, University of Twente, P.O. Box 217, 7500 AE Enschede, The Netherlands

**Keywords:** polyphosphoesters, hemocompatibility, blood coagulation, RBC interaction, platelet activation, poly(ethylene glycol), biodegradable polymers

## Abstract

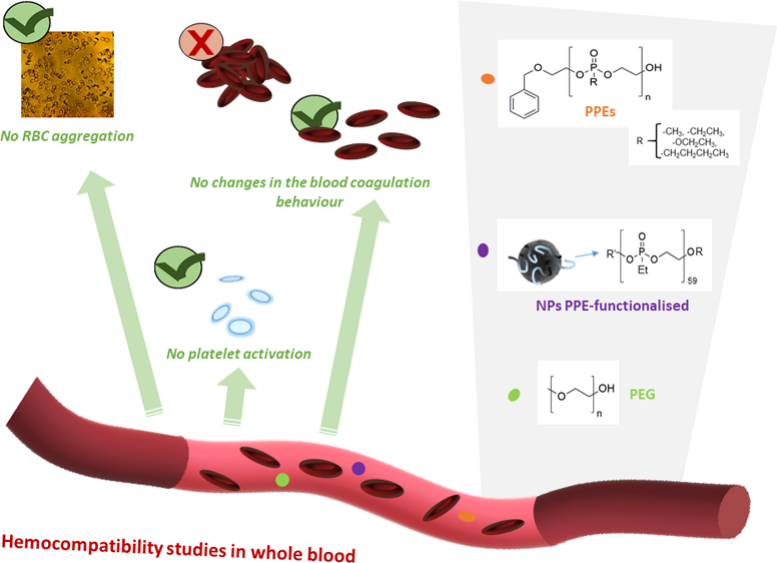

Polyphosphoesters (PPEs) are a class of versatile degradable polymers. Despite the high potential of this class of polymers in biomedical applications, little is known about their blood interaction and compatibility. We evaluated the hemocompatibility of water-soluble PPEs (with different hydrophilicities and molar masses) and PPE-coated model nanocarriers. Overall, we identified high hemocompatibility of PPEs, comparable to poly(ethylene glycol) (PEG), currently used for many applications in nanomedicine. Hydrophilic PPEs caused no significant changes in blood coagulation, negligible platelet activation, the absence of red blood cells lysis, or aggregation. However, when a more hydrophobic copolymer was studied, some changes in the whole blood clot strength at the highest concentration were detected, but only concentrations above that are typically used for biomedical applications. Also, the PPE-coated model nanocarriers showed high hemocompatibility. These results contribute to defining hydrophilic PPEs as a promising platform for degradable and biocompatible materials in the biomedical field.

## 1 Introduction

Polyphosphoesters (PPEs) belong to the few polymer classes that enable the synthesis of well-defined and degradable water-soluble polymers.^1,2^ The presence of pentavalent phosphorous in the main chain allows for tuning the polymer properties (e.g., hydrophilicity, crystallinity, degradability, thermal stability, and so forth) by varying the lateral group, providing the polymers with high versatility. To date, several studies have reported the hydrolytic and enzymatic degradation of PPEs in vitro,^3,4^ and PPE-containing (co)polymers were reported as noncytotoxic against different cell lines even at high concentrations.^1^ In addition, hydrophilic PPE-coated nanocarriers exhibit a so-called “stealth effect,” and their cellular uptake can be controlled depending on the polymer hydrophilicity.^5^ Based on such results, PPEs have been proposed for different biomedical applications, for example, protein–polymer conjugates,^6−8^ nanocarriers loaded with drugs,^9^ antimicrobial agents,^10^ gene vectors,^11^ or hydrogels for tissue engineering.^12^ In many applications, PPEs are proposed as biodegradable alternatives to poly(ethylene glycol) (PEG),^1,13,14^ which is nowadays the most used water-soluble polymer in nanomedicine to prolong the drug lifetime and efficiency.^15^ In this context, the evaluation of PPE blood compatibility is an important step to translate these in vitro results to in vivo applications. However, there is limited information available on the blood compatibility of this class of polymers. In a study, Murgia and co-workers evaluated the hemolysis and the complement system activation by cubosomes, that is, lyotropic nanoparticles with a cubic internal nanostructure that were self-assembled from poly(propylene oxide)-*block*-poly(methyl ethylene phosphate). Lower cytotoxicity compared to the commonly used Pluronic F127 analogues was detected.^16^ A systematic blood compatibility study of different PPEs has not been reported to date. Here, we report the hemocompatibility of a set of PPEs with various molar masses and different hydrophilicities, as well as PPE-coated model nanocarriers. The results were compared to those of PEG, aiming at detecting any difference in the hemocompatibility in view of future medical applications.

## 2 Experimental Section

### 2.1 Materials

### 2.2 Polymers Synthesis and Features (Samples #1–4)

Polyphosphoesters (PPE) synthesis was performed via ring-opening polymerization, as described previously.^7,17^ After the synthesis, the polymers were dialyzed for 24 h in clinical water (sterile and endotoxin-free). The endotoxin level was tested by the Limulus amebocyte lysate assay (kit from Thermofisher), and only the samples with an endotoxin level below 0.5 U/mL were taken into consideration for further studies. For the determination of the polymer molecular weight, gel permeation chromatography (GPC) was performed in *N,N*′-dimethylformamide (DMF; containing 0.25 g/L of lithium bromide as an additive) using an Agilent 1100 Series as the integrated instrument, including a PSS GRAM columns (1000/1000/ 100 g), a UV detector (280 nm), and an RI (refractive index) detector at a flow rate of 1 mL/min at 60 °C. Calibration was carried out using PS standards provided by the Polymer Standards Service. ^1^H and ^31^P {H}-NMR spectra were acquired at 298.3 K with a Bruker AVANCE III 300 MHz spectrometer. The spectra were calibrated against the solvent signal and analyzed using MestReNova 9.0.0 from Mestrelab Research S.L. The PPEs’ features and a representative ^1^H NMR spectrum are reported in the Supporting Information, Section 1.

### 2.3 Preparation of PPEylated Polystyrene Model Nanocarriers (Sample #6)

Polystyrene nanoparticles (PS-NPs) BODIPY-labeled, amino-functionalized, CTMA-Cl stabilized were synthesized and characterized following the procedure, and the methodologies have been described in a previous study.^5^ (*D*_h_ (DLS): 118 nm; −NH_2_ groups per particle: 20,000; solid content: 1.0%.). The polymer poly(ethyl ethylene phosphonate) ω-functionalized with 4-(maleinimido) phenyl isocyanate (PEtEP-MAL) was synthesized and characterized as specified previously.^5^ (Yield: 98%; *M*_n_ (NMR): 8400 g/mol (59 repeated units); 72.5% of functionalization; *Đ* = 1.4). Nanoparticle functionalization was performed as described: 72 μL of pyridine were added to 3 mL of PS-NP dispersion (1% wt, 3.3 × 10^13^ particles, 1.2 × 10^–6^ mol NH_2_ groups) to reach pH 8.3. The dispersion was stirred at 500 rpm for 20 min at room temperature. Then, 2.5 eq. of PEtEP-MAL (with respect to the −NH_2_ groups, 2.9 × 10^–3^ mmol) was dissolved in 1 mL of sterile Millipore water and added dropwise to the dispersion. The reaction was stirred for 24 h at room temperature and 500 rpm to ensure full conversion. The dispersion was purified by repeated centrifugation (3 × 1 h, 30,000 g). Each time, the supernatant was removed, and the pellet was redispersed in sterile Millipore water. After the last centrifugation, the dispersion was adjusted to 1 wt. % with sterile Millipore water.

Characteristics of PPE-functionalized PS-NPs, sample 6: *D*_h_ (DLS): 122 nm; ξ-potential: −30.1 ± 6.7 mV; solid content: 1.0 wt. %; number of polymers attached to each particle: ca. 4000 (calculated by ^1^H NMR spectroscopy, as previously described^5^). Pictures of sample 6 observed by optical microscopy are reported in the Supporting Information, Section 1.

### 2.4 Blood Sample Preparation

Blood was collected from healthy consenting donors in a 3.2% sodium-citrated vacutainer tube at the Centre for Blood Research, University of British Columbia, Canada. For serum preparation, whole blood was collected in a nonanticoagulated vacutainer tube. Platelet-rich plasma (PRP) was prepared by centrifuging citrated whole blood samples at 150×*g* for 15 min in an Allegra X-22R centrifuge (Beckman Coulter, Canada). Platelet-poor plasma (PPP) was prepared by centrifuging citrated whole blood samples at 2000×*g* for 20 min. Serum was prepared by centrifuging nonanticoagulated whole blood samples at 2000×*g* for 20 min after resting 30 min at room temperature to clot. The PPE samples were dissolved in isotonic saline 0.9% solution. Both the PPE samples and all the reagents were preincubated at 37 °C for 20 min before mixing. PPE stock solutions at 1, 5, and 10 mg/mL were prepared in 0.9% isotonic saline solution. The PPE samples were mixed in a 1/10 ratio with plasma or whole blood to achieve final polymer concentrations of 0.1, 0.5, and 1 mg/mL.

### 2.5 Platelet Activation by Flow Cytometry

Fresh blood from three different donors was collected, and PRP was prepared. Ten microliters of stock PPE samples (in duplicate) were incubated with 90 uL of PRP in 1.5 mL polystyrene tube for 1 h at 37 °C. A small aliquot of the platelet suspension (5 μL) was mixed with 2.5 μL mouse antibody monoclonal CD62P PE in 45 μL of autologous plasma pH 7.4. Platelet activation was analyzed by flow cytometry (BD, FACS Canto II), and 10,000 events were recorded for each run. The mouse monoclonal anti-CD42a-FITC human antibody was incubated also in the same proportion with the PRP to confirm the presence of the platelet in the flow gate. The 1 mM TRAP6 was used as a positive control for platelet activation; 45 μL of PRP were incubated with 5 μL of thrombin receptor-activated peptide (TRAP) for 15 min at room temperature.

### 2.6 Activated Partial Thromboplastin Time and Prothrombin Time Analysis

The activated partial thromboplastin time (APPT) and prothrombin time (PT) assay were used to monitor the effect of PPE on blood coagulation. The polymer stock solution (25 μL) was mixed with the PPP (225 μL) at 37 °C. Saline solution was used as the normal control. Coagulation initiation reagents actin FSL and Innovin were used for APTT and PT analyses, respectively. Triplicate samples were tested on the STart4 coagulometer (Diagnostica Stago, France) using plasma from three separate donors, and the average ± SD was reported.

### 2.7 Rotational Thromboelastometry Analysis

The rotational thromboelastometry (ROTEM) system (ROTEM-*delta*) was used for this coagulation study. Sodium citrate anticoagulated whole blood fresh collected (within 15 min) (360 μL) was mixed with the saline control or stock PPE solutions (40 μL) (9:1 v/v). The blood–polymer mixture (340 μL) was transferred into the ROTEM cup. The coagulation was initiated by adding 20 μL of 0.2 M calcium chloride solution. The ROTEM clot’s physical and kinetic properties were analyzed for three donors.

### 2.8 Red Blood Cell Aggregation and Hemolysis

Citrate anticoagulated whole blood or red cells (10% hematocrit) washed in phosphate buffer were used for these studies. The PPE sample (20 μL) or saline control was incubated with 180 μL of red blood cell samples for 1 h at 37 °C. For the aggregation study, the cells were fixed with 2% glutaraldehyde in saline (2 h incubation at room temperature). The blood cell morphology was assessed using a bright-field light microscope (Zeiss Axioskop 2 Plus, 40× magnifications) with a digital microscope camera (AxioCam ICC 1, Carl Zeiss Microimaging Inc.) attached to it. For the hemolysis study, RBCs incubated with distilled H_2_O were used as the positive control (100% lysis). The percentage of RBC lysis was measured on a 96-well plate Spectra Max 190 spectrophotometer using the ferricyanide–cyanide (Drabkin’s) method. Two microliters of the RBC/PPE mixture were added to 298 μL of Drabkin’s solution, and the optical density (OD) at 540 nm of the solution was measured. The remaining RBC/PPE mixture was centrifuged, and 50 μL of the supernatant was added to 250 μL of Drabkin’s solution. The OD of the solution was measured using Drabkin’s reagent as a blank. The percentage of lysed cells was calculated from the ratio of hemoglobin present in the supernatant and the total hemoglobin.

## 3 Results and Discussion

### 3.1 Synthesis and Characterization of PPEs and Nanocarriers

**Figure 1:**
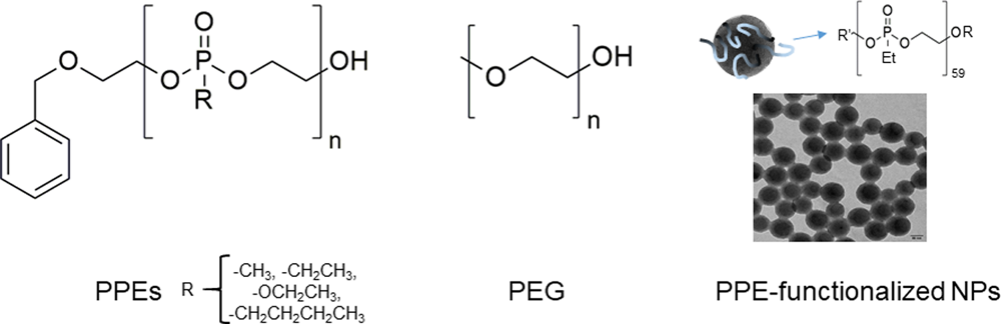
Set of samples analyzed in this work.

In the present work, a set of PPEs (Figure 1) was synthesized via ring-opening polymerization of cyclic phosphates or phosphonates, and the PPE polymers were characterized for their purity, molar mass, and molar mass dispersity. The PPE-coated nanocarriers were prepared by aza-Michael addition between the amino group on the polystyrene surface and poly(ethyl ethylene phosphonate) (PEtEP), which was previously ω-functionalized with commercial 4-(maleinimido)phenyl isocyanate (Figure 1). More details about the samples and their main features are shown in Table 1.

**Table 1:** Library of Samples Analyzed in This Work

type of sample	sample	name	features
PPEs	**1**	poly(methyl ethylene phosphonate (PMeEP)	R = −CH_3_; *M*_n_: 10 kDa; DP_n_: 75
	**2a**	poly(ethyl ethylene phosphonate) (PEtEP)	R = −CH_2_CH_3_; *M*_n_: 7 kDa; DP_n_: 50
	**2b**	poly(ethyl ethylene phosphonate) (PEtEP)	R = −CH_2_CH_3_; *M*_n_: 10 kDa; DP_n_: 75
	**2c**	poly(ethyl ethylene phosphonate) (PEtEP)	R = −CH_2_CH_3_; *M*_n_: 26 kDa; DP_n_: 190
	**3**	poly(ethyl ethylene phosphate) (PEEP)	R = −OCH_2_CH_3_; *M*_n_: 7.2 kDa; DP_n_: 50
	**4**	poly(ethyl ethylene-*co* butyl ethylene phosphonate) (PEtBuEP)	R = −CH_2_CH_3_, −CH_2_CH_2_CH_2_CH_3_*M*_n_: 10 kDa; DP_n_: 71; 36 (−CH_2_CH_3_) and 31 (−CH_2_CH_2_CH_2_CH_3_)
PEG	**5a**	polyethylene glycol (PEG)	*M*_n_: 8 kDa; DP_n_: 133
	**5b**	polyethylene glycol (PEG)	*M*_n_: 20 kDa; DP_n_: 333
	**5c**	polyethylene glycol (PEG)	*M*_n_: 35 kDa; DP_n_: 583
PPE-functionalized PS-NPs	**6**	PS nanoparticles functionalized with PEtEP	PEtEP: R = −CH_2_CH_3_; *M*_n_: 8.4 kDa; DP_n_: 59; number of polymers attached to each particle: 4000

### 3.2 Blood Compatibility Studies of PPEs

The influence of polymers on blood coagulation, as well as the interaction with platelets and RBCs were recently investigated in various polymers proposed for biomedical applications.^18−21^ In the current work, hemocompatibility assays were performed on the samples using fresh blood collected from consented donors at the University of British Columbia.

#### 3.2.1 Influence of Blood Coagulation

**Figure 2:**
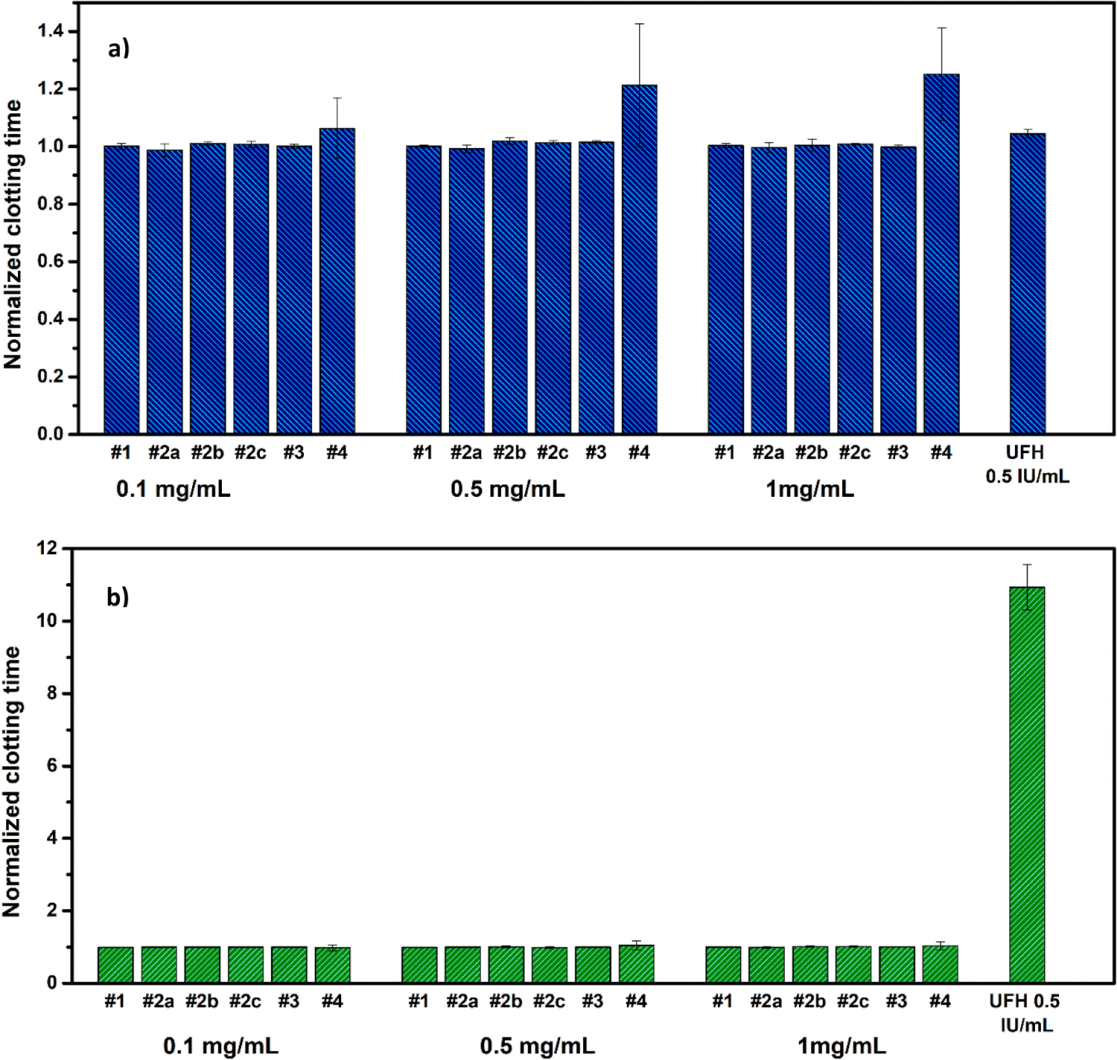
Effect of PPE polymers on (a) PT and (b) APTT at different concentrations (0.1, 0.5, and 1 mg/mL). The clotting time was normalized for the saline control run for each sample (33.2 ± 2.8 s for APTT and 9.8 ± 1.1 s for PT analysis). Unfractionated heparin (UFH) was used as a control. The data have been obtained by analyzing the blood of three different donors (*N* = 3).

The influence of PPEs on blood coagulation was investigated by a number of assays including the measurement of plasma clotting times and whole blood clotting in human blood. The pro- or anticoagulant nature of the PPE polymers was measured using two clinical coagulation assays: PT (Figure 2a) and APTT (Figure 2b) in human plasma. The data showed no significant changes in the blood coagulation behavior at any of the concentrations tested (0.1 to 1 mg/mL final concentration) with respect to the buffer-incubated plasma control, differently from the positive control (heparin, UFH), which showed an anticoagulant effect in the APTT assay.

**Figure 3:**
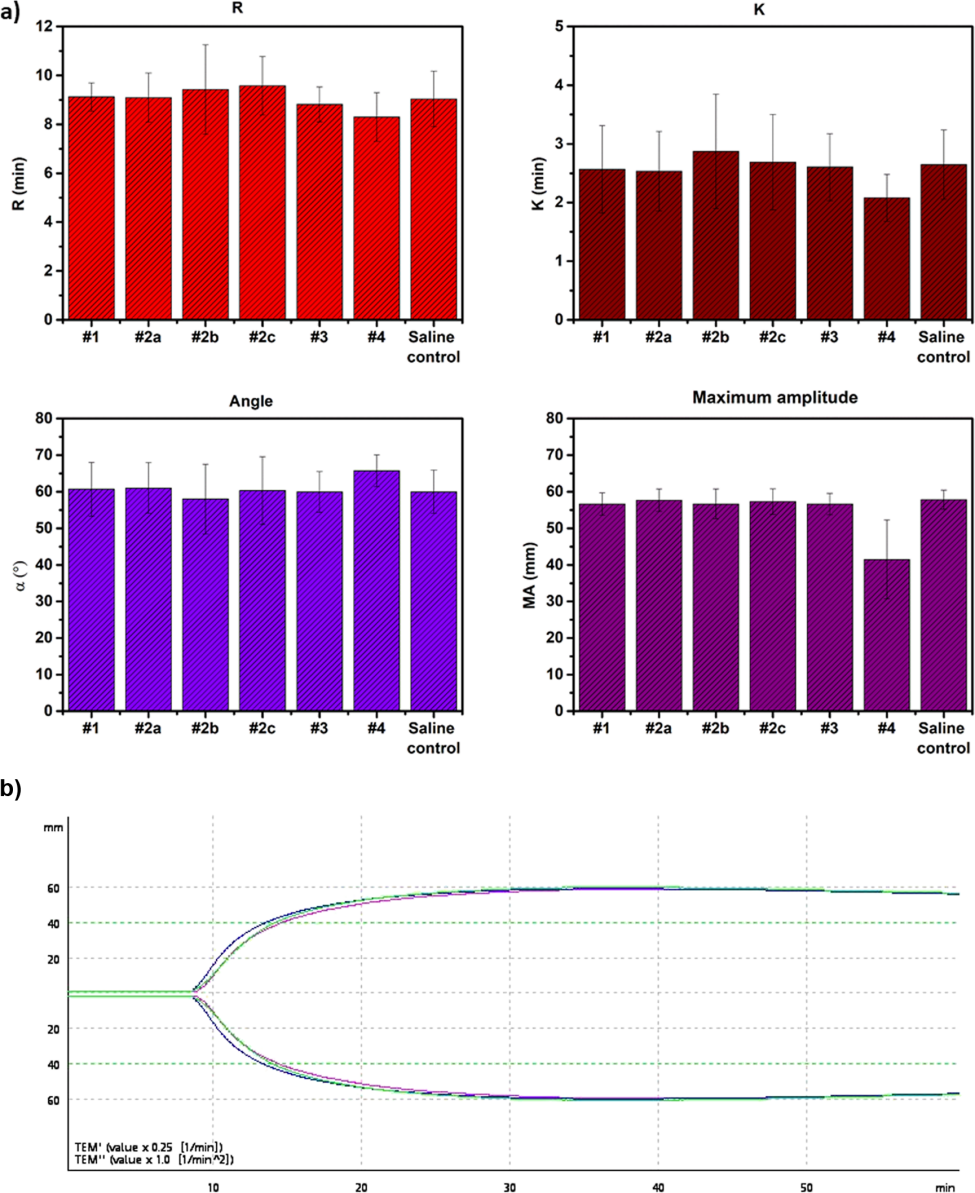
Whole blood coagulation studied using ROTEM in the presence of PPEs. (a) Reaction time (R), time for the first significant clot formation, K (achievement of certain clot firmness, angle (kinetics of clot development), and MA (maximum amplitude–maximum strength of clot). (b) ROTEM profile of sample #1 at 1 mg/mL (as made (blue), 2 weeks (green), and saline control (pink)).

To further investigate the influence of PPEs on whole blood coagulation, ROTEM was used.^22^ Whole blood samples (*N* = 3, independent donors) were incubated with different PPEs at 1 mg/mL concentration, and the clotting parameters were measured, including the “R” time, “K” values, and angles and maximum clot firmness (MA) (Figure 3a). We also measured the influence of PPEs incubated in buffer for 2 weeks and then used for ROTEM measurements to understand whether polymer degradation influences blood coagulation. The ROTEM profile of sample #1 is shown in Figure 3b, and the others are reported in the Supporting Information, Figure S2. As shown in the figure, the PPEs did not modify any of the ROTEM parameters, even in their degraded form (measurements of samples after 2 weeks of incubation in buffer), suggesting that the PPEs do not influence the whole blood clot formation. Only sample #4 at a concentration of 1 mg/mL showed an unusual ROTEM profile (Supporting Information Figure S2), similar to that shown by amphiphilic polymers such as poly(*N*-isopropylacrylamide).^18^ Nevertheless, it is important to note that this sample at lower concentrations did not modify the blood clotting profile.

#### 3.2.2 Platelet Interaction with PPEs

**Figure 4:**
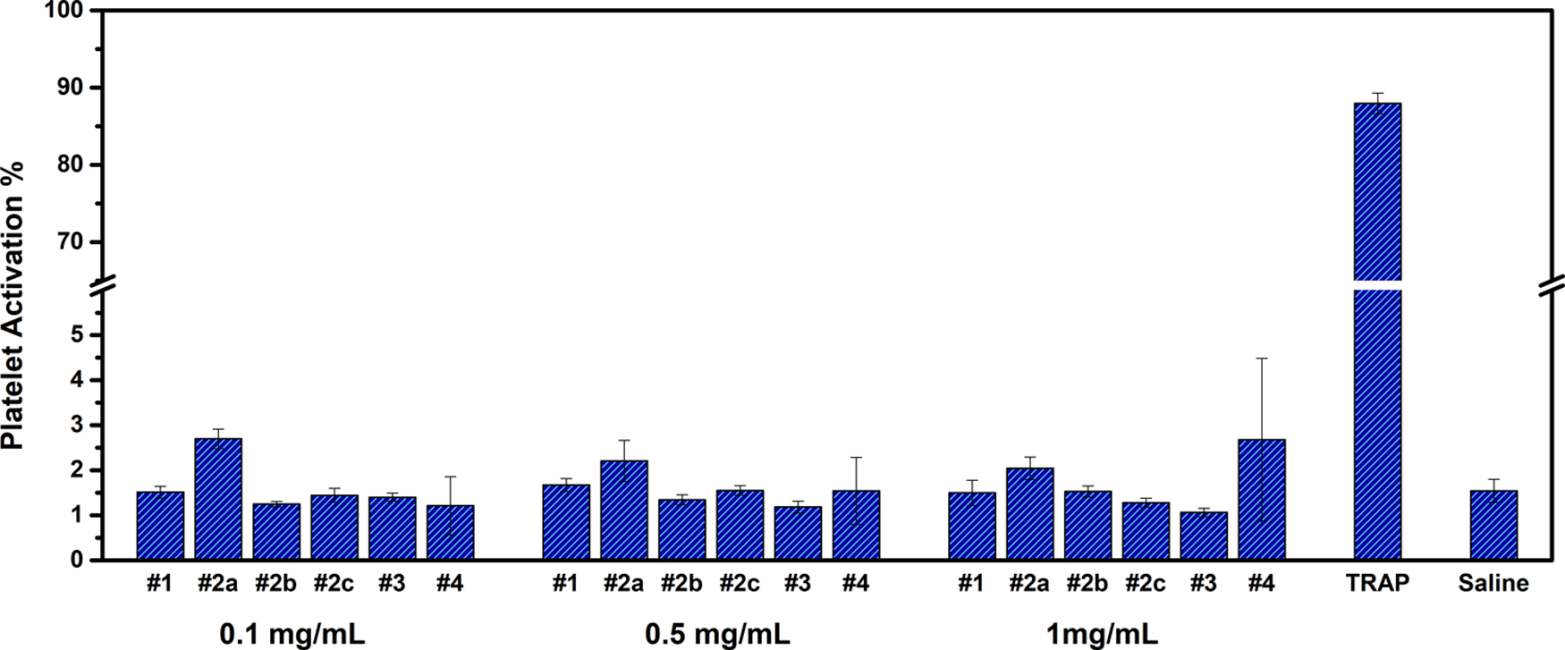
Platelet activation in the presence of the PPE polymers at different concentrations, observed by measuring the expression of the activation marker CD62P on the surface of platelets using flow cytometry analysis. Saline solution and TRAP have been used as, respectively, negative and positive controls for the experiment.

In support of the whole blood clotting profile previously shown, we next investigated the interaction of platelets with PPEs by measuring the platelet activation using a flow cytometer in PRP. We measured the activation marker CD62P on the platelets’ surface using an anti-CD62P antibody. The data obtained are reported in Figure 4. We observed that the PPE samples did not show any significant platelet activation at any concentration, confirming the absence of unfavorable polymer interactions with the platelets. The interaction was almost unaffected by changes in the PPE polymer hydrophilicity (polymers #1, #2b, #4) or molar mass (polymers #2a–c).

#### 3.2.3 RBC Aggregation and Hemolysis in the Presence of PPEs

**Figure 5:**
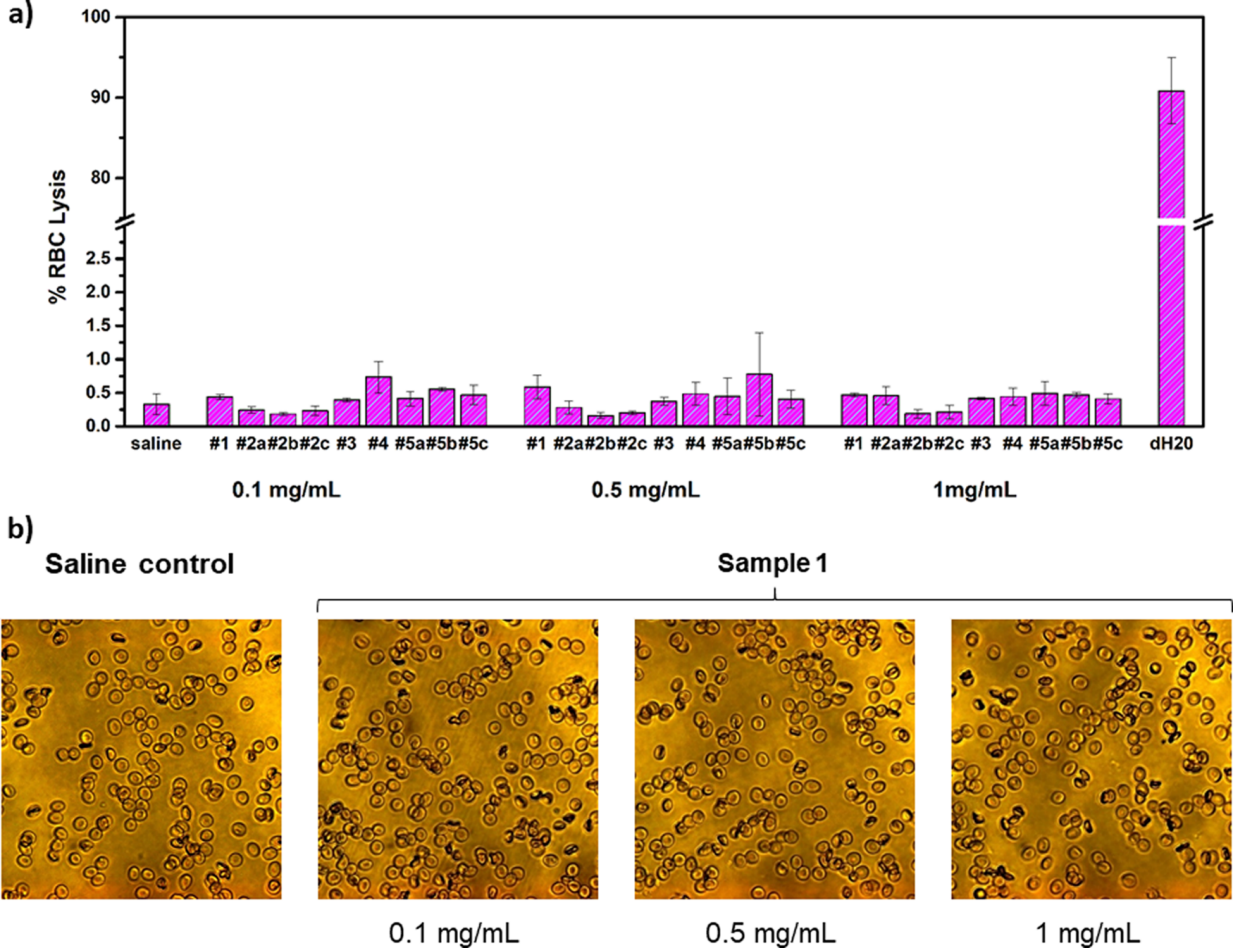
(a) Lysis of RBC after incubation with the tested PPE polymers at different concentrations (0.1, 0.5, and 1 mg/mL); saline solution and deuterated water (D_2_O) were used as, respectively, negative and positive controls for the experiment. (b) Representative images obtained by optical microscopy, showing RBC aggregation in the presence of sample #1 (magnification: 40×). The sample does not show any detectable aggregation at all the concentrations tested (0.1, 0.5, and 1 mg/mL), in comparison to the saline control shown on the left. The optical micrographs recorded for the other samples are reported in the Supporting Information, Figure S3.

Furthermore, we investigated the interaction of PPEs with RBCs by measuring their lysis in whole blood and by observing the RBC aggregation by optical microscopy.^21^ The PPE behavior was compared to PEG chains with different molar masses (8, 20, or 35 kDa, see Table 1), with the aim to evaluate the potentialities of PPEs as PEG substitutes in the biomedical field. The results are reported in Figure 5.

Linear hydrophilic polymers (e.g., dextran ≥40 kDa) are known to promote RBC aggregation by adsorbing to their surface and forming bridges between the cells.^23^ On the contrary, PPEs induced negligible cells’ lysis at all the concentrations tested (Figure 5a), showing comparable results to PEG samples (#5a–c). Moreover, we did not detect any RBC aggregation or crenation by optical microscopy in the PPEs (Figure 5b), except for sample #4 at 1 mg/mL concentration, which showed slight aggregation in the more concentrated solution (1 mg/mL, see the Supporting Information Figure S3). This behavior, in agreement with the unusual ROTEM profile observed above, was probably related to the increased hydrophobicity of sample #4, given by the presence of butyl residues in the polymer lateral chain (see Figure 1). We assume that RBC interactions with PPEs depend on the PPE functionalization and hydrophilicity, especially at high concentrations. However, we did not observe any dependence on the polymer molar mass.

### 3.3 Blood Compatibility of PPE-Coated Nanocarriers

**Figure 6:**
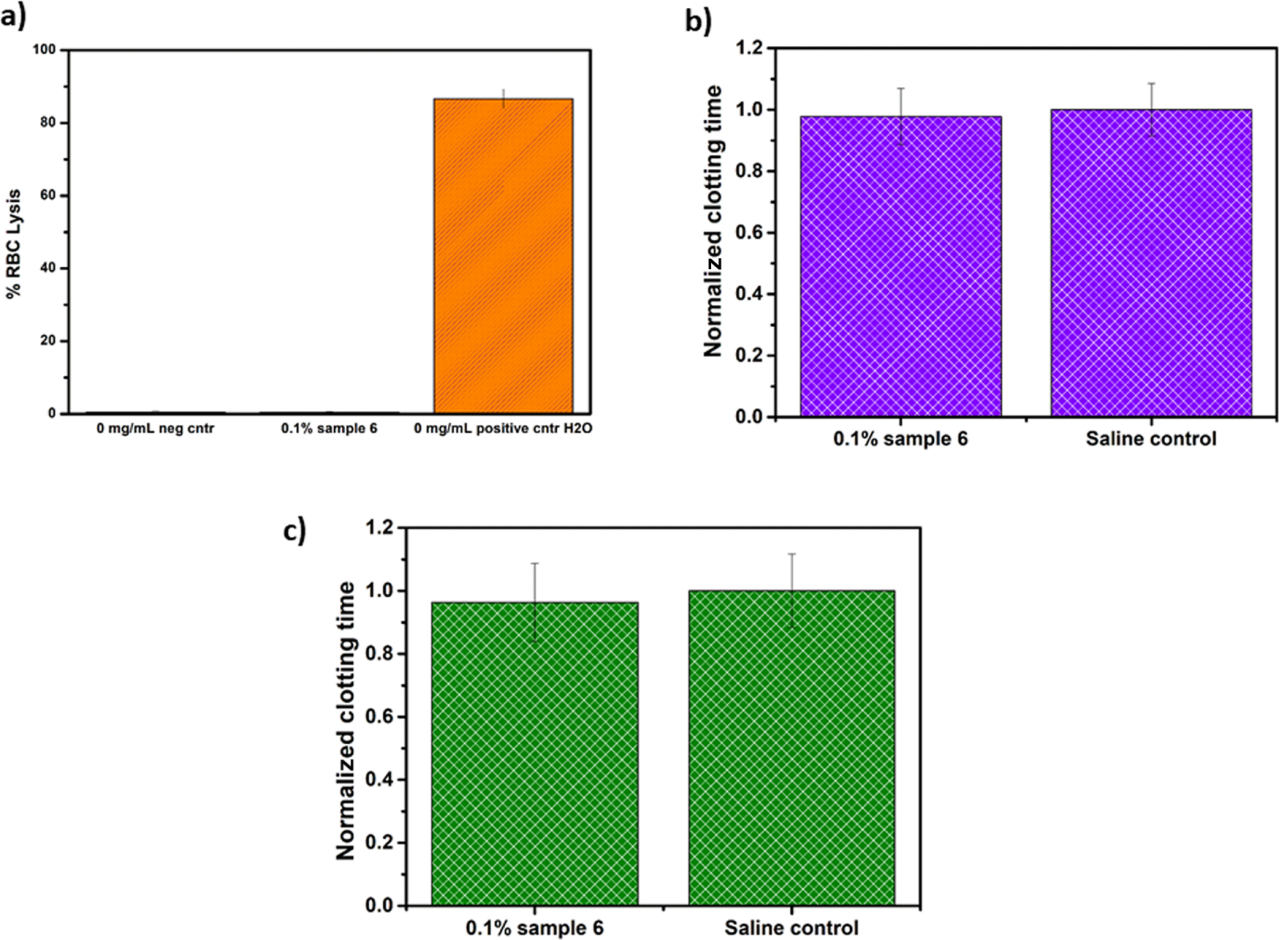
(a) Lysis of RBCs after incubation with sample #6. Effect of sample #6 on (b) APTT and (c) PT. The clotting time was normalized for the saline control run for each sample. The data have been obtained by analyzing the blood of three different donors (*N* = 3).

Having obtained promising results for water-soluble PPEs, we also performed first investigations on polystyrene nanoparticles (NPs) coated with PPEs as model nanocarriers for drug loading in biomedical applications. Such model nanocarriers were studied before with respect to cellular uptake and protein adsorption and showed stealth properties similar to their PEGylated analogues.^24^ The functionalized NPs exhibited good antithrombogenicity, absence of RBC lysis, and unaltered blood viscoelastic parameters, calculated by ROTEM analysis (Figure 6 and Figure S4), even though small light crenation was observed in the blood cells by optical microscopy (Figure S3). Overall, the data set acquired confirmed the high potential of PPE-functionalized NPs for biomedical applications, as at this preliminary stage, they showed satisfactory hemocompatibility data. Further analyses, for a deeper understanding of their interaction with different blood cells and proteins, were beyond the scope of this work and will be the object of future investigations.

## 4 Conclusions

We report a systematic study of blood compatibility for a set of hydrophilic PPEs with different hydrophilicities and molar masses. We found high hemocompatibility for all the investigated PPE-samples, which did not alter the blood coagulation profile, and induced negligible platelet activation, RBC lysis, or aggregation. Changes in the molar mass (samples #2a–c) did not influence polymer hemocompatibility. The more hydrophobic copolymer (sample #4) presented some changes in the whole blood clot strength at the highest concentration; nonetheless, it was still very compatible at the concentrations usually needed for biomedical applications (<1 mg/mL). The conjugation of PPEs to polystyrene nanoparticles, proposed as model drug nanocarriers, at a first glance, did not alter their hemocompatibility, even though small light crenation of the RBCs incubated with the NPs 0.1% w/w was observed by optical microscopy. These results underline that hydrophilic PPEs are promising, biodegradable substitutes to currently used PEG, which might cause side effects in some patients.^15,25^ We believe that the data presented herein will further stimulate the use of PPEs in biomedical and translational studies for fully degradable drug formulations without negative immune responses.

## Supporting Information Available

Sample characterization (polymer features, representative NMR spectrum, and PPE-functionalized NPs observed by optical microscopy); ROTEM profiles of samples #2–4; RBC aggregation of samples #2–5 observed by optical microscopy; and blood coagulation parameters obtained by ROTEM analysis on PPE-functionalized nanoparticles (sample #6) (PDF)
