# Supplementary material for: Blood Compatibility of Hydrophilic Polyphosphoesters
Source: ACS Appl Bio Mater. 2022 Feb 24;5(3):1151–8. doi: 10.1021/acsabm.1c01210 (PMC8941511; doi:10.1021/acsabm.1c01210)
Supplement: Supplementary file 1 — mt1c01210_si_001.pdf [file mt1c01210_si_001.pdf]

SUPPORTING INFORMATION

Blood compatibility of hydrophilic polyphosphoesters

Chiara Pelosi,[1] Iren Constantinescu,[2] Helena H. Son,[2] Maria Rosaria Tinè,[1] Jayachandran N. Kizhakkedathu,[2,3,] Frederik R. Wurm[4,*]*

1: Dipartimento di Chimica e Chimica Industriale, Università di Pisa, Via Moruzzi 13, 56120 Pisa (PI), Italy.

2: Center for Blood Research, Life Sciences Centre, Department of Pathology and Laboratory Medicine, University of British Columbia, 2350 Health Sciences Mall #4302, Vancouver, BC V6T 1Z3, Canada.

3. School of Biomedical Engineering, University of British Columbia, 2350 Health Sciences Mall #4302, Vancouver, BC V6T 1Z3, Canada.

4: Sustainable Polymer Chemistry (SPC), Department of Molecules and Materials, MESA+ Institute for Nanotechnology, Faculty of Science and Technology, University of Twente, P.O. Box 217, 7500 AE Enschede (Netherlands).

*Corresponding authors e-mails: f.r.wurm@utwente.nl jay@pathology.ubc.ca

TABLE OF CONTENTS:

1. SAMPLES' CHARACTHERIZATION
2. HEMOCOMPATIBILITY ANALYSES

3. REFERENCES

1. SAMPLES' CHARACTHERIZATION

Table S1: Features of the synthesized polymers.

Name	Description	M_n (NMR) (g/mol)[a]	Molar mass dispersity $Đ^b$
1	Poly(methyl ethylene phosphonate (PMeEP)	10000	1.3
2a	Poly(ethyl ethylene phosphonate) (PEtEP)	7000	1.1
2b	Poly(ethyl ethylene phosphonate) (PEtEP)	10000	1.2
2c	Poly(ethyl ethylene phosphonate) (PEtEP)	26000	1.1
3	Poly(ethyl ethylene phosphate) (PEEP)	7200	1.2
4	Poly (ethyl ethylene – *co*- butyl ethylene phosphonate) (PEtBuEP)	10000	1.2

a: Polymer molar mass, calculated by ^{1}H NMR as previously described.[1] b: Molar mass dispersity, calculated by GPC and defined as $Đ = M_w/ M_n$.

Representative ^{1}H-NMR spectrum of sample 1: ^{1}H NMR (DMSO-d_6, 300 MHz): δ [ppm] 7.39-7.25 (m, 5H, Ar), 4.83 (s, 1H, terminal -OH), 4.53 (s, 2H, Ar-CH_2), 4.30-3.98 (m, 304 H, backbone O-CH_2-CH_2-O), 1.52-2.05 (d, 228 H, —CH_3 side chain), ^{31}P {H}-NMR (DMSO-d_6, 121 MHz): δ [ppm] = 31.85.

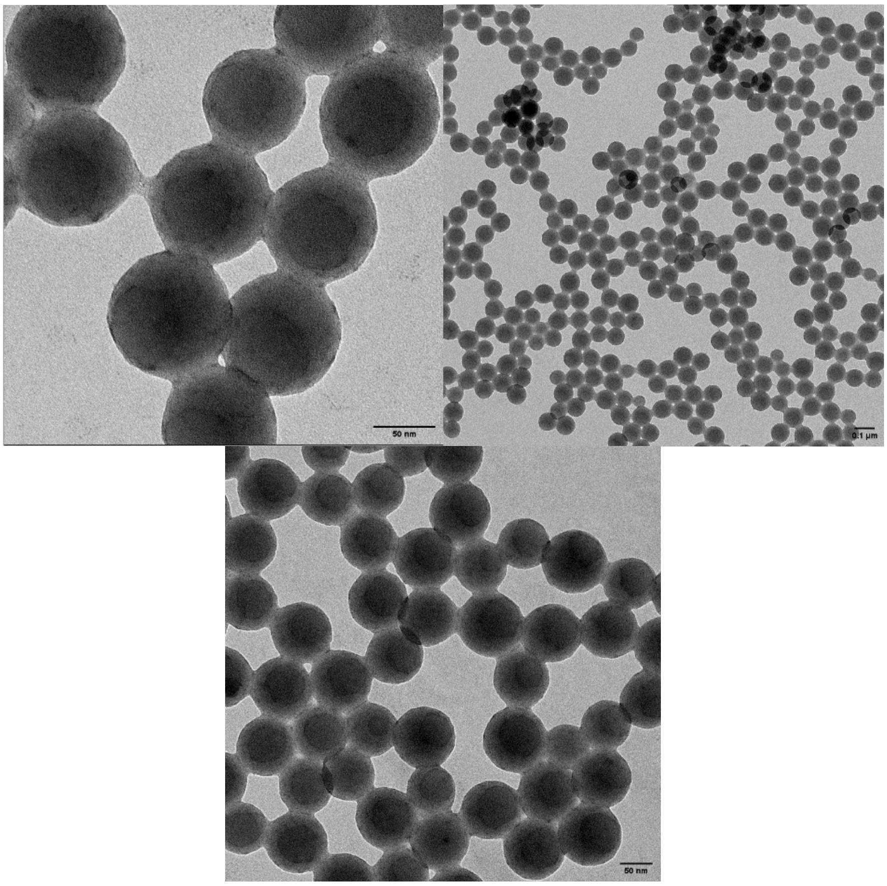

Figure S1: PPE-functionalised PS-NPs (sample 6) observed by optical microscopy.

2. HEMOCOMPATIBILITY ANALYSES

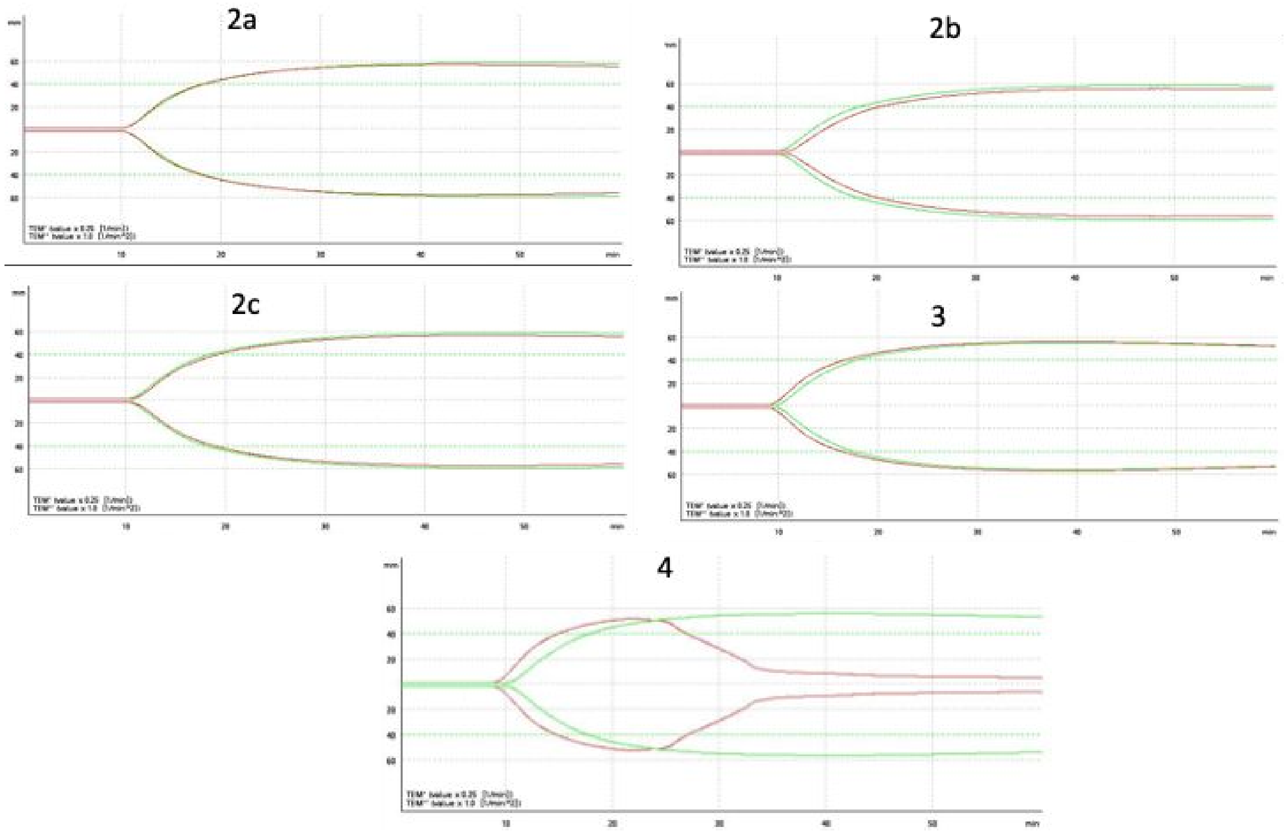

Figure S2: ROTEM profiles for samples #2-4 in whole blood at 1 mg/mL (final concentration). Representative profiles are shown. Three independent experiments (N=3) were performed. Green line is for saline control (for all samples) and red line is for different samples.

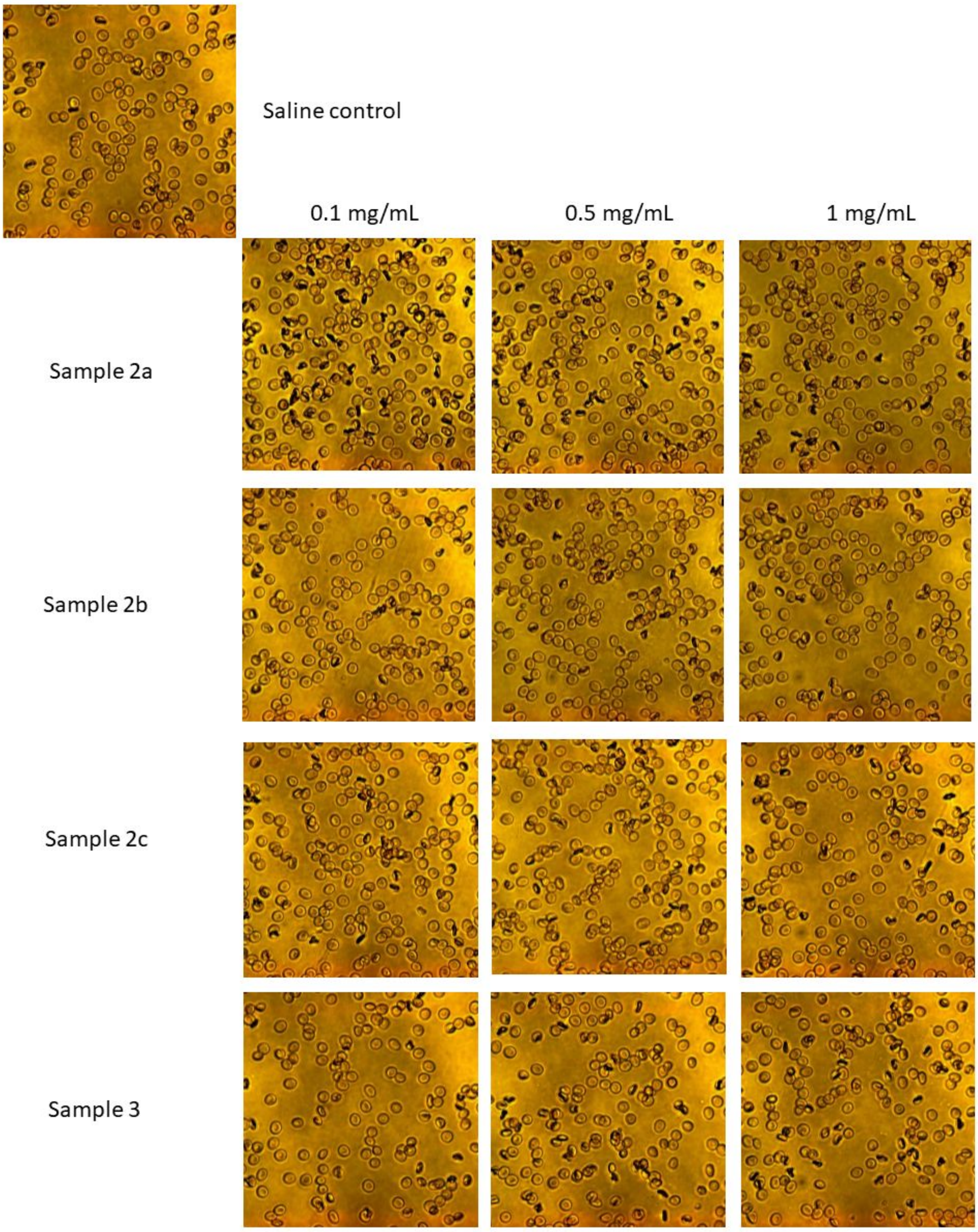
Saline control
0.1 mg/mL
0.5 mg/mL
1 mg/mL
Sample 2a
Sample 2b
Sample 2c
Sample 3

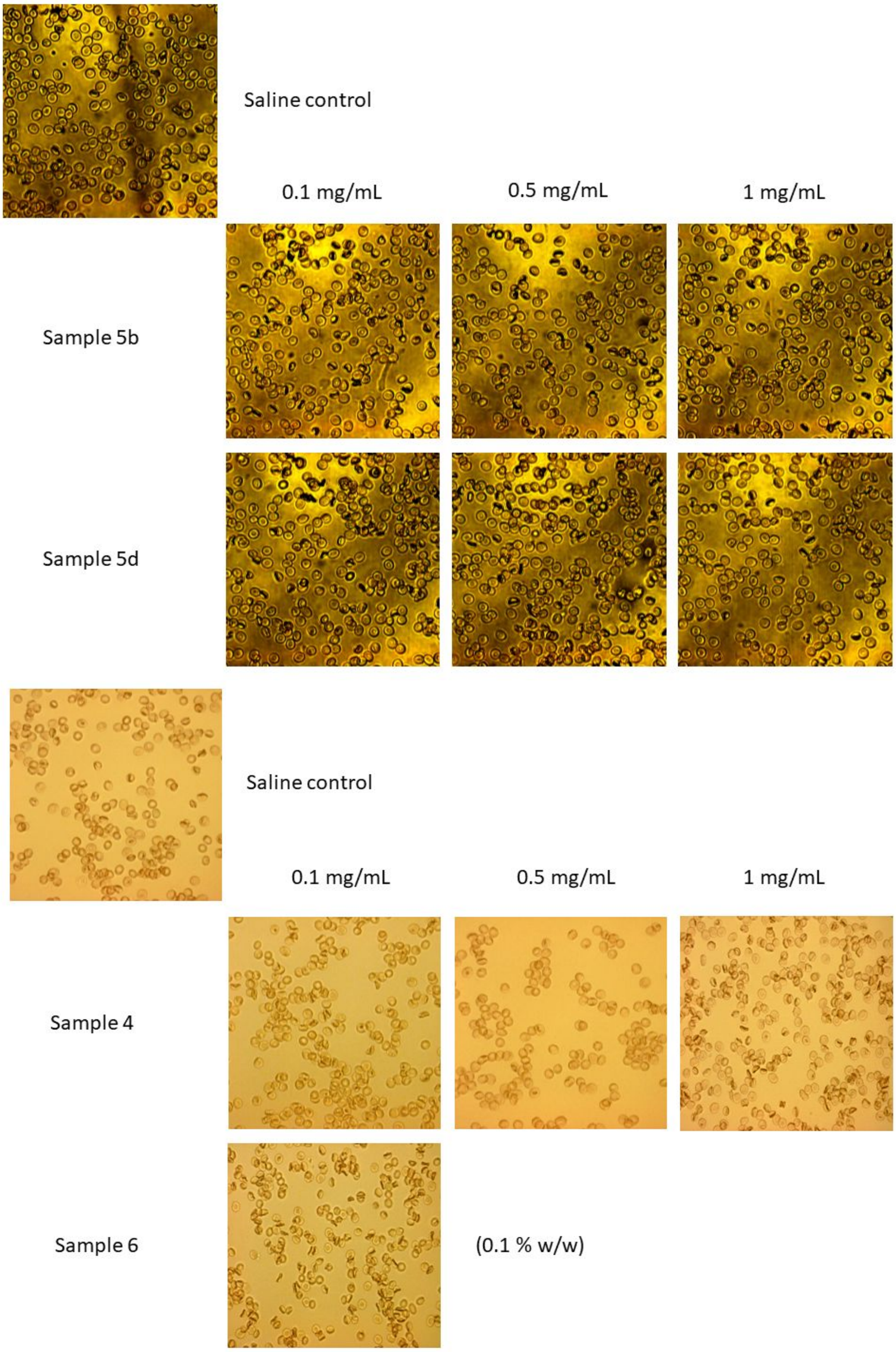

Figure S3: RBC aggregation of samples #2-5 observed by optical microscopy (magnification: 40x). The samples do not show any detectable aggregation at all the concentrations tested (0.1, 0.5, 1mg/mL), except for sample #4 at 1 mg/mL concentration, which show slight aggregation.

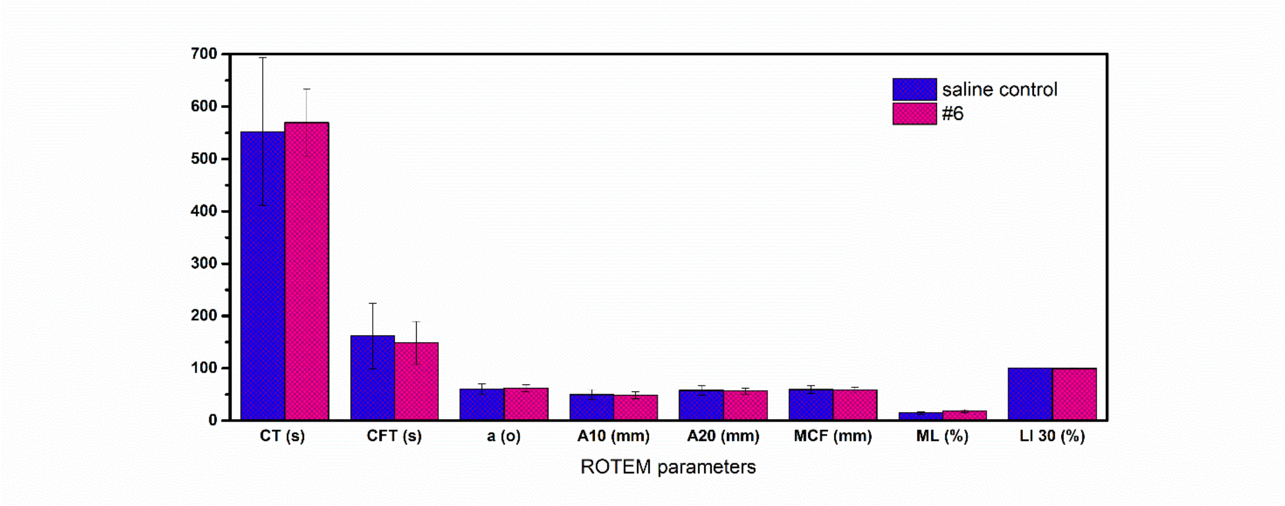

Figure S4: Blood coagulation studied using ROTEM in presence of PPE-coated PS nanoparticles (sample #6) compared to the saline control. The data is plotted as shown. In comparison to the saline control, there was no difference in ROTEM parameters for sample #6.

3. REFERENCES

(1) Steinbach, T.; Becker, G.; Spiegel, A.; Figueiredo, T.; Russo, D.; Wurm, F. R. Reversible Bioconjugation: Biodegradable Poly(Phosphate)-Protein Conjugates. *Macromol. Biosci.* **2017**, *17*, 1600377. https://doi.org/10.1002/mabi.201600377.
